# Screening for Media Use in the Emergency Department Among Young Australians: Cross-sectional Study

**DOI:** 10.2196/42986

**Published:** 2023-05-15

**Authors:** Pravin Dullur, Joanne Joseph, Antonio Mendoza Diaz, Ping-I Lin, Rajeev Jairam, Rhian Davies, Anne Masi, Boaz Shulruf, Valsamma Eapen

**Affiliations:** 1 Discipline of Psychiatry and Mental Health University of New South Wales Sydney Australia; 2 Infant, Child and Adolescent Mental Health Services South Western Sydney Local Health District Liverpool Sydney Australia; 3 Discipline of Mental Health Western Sydney University Sydney Australia; 4 Medical Education Faculty of Medicine University of New South Wales Sydney Australia

**Keywords:** internet, mental illness, overuse, problematic internet behaviors, emergency department, screening, adolescent, youth, internet use, mental health, technology use, young people, internet behavior

## Abstract

**Background:**

Research on problematic internet use has largely adhered to addiction paradigms, possibly impeding the identification of specific internet behaviors related to psychopathology. This study presents a novel approach to screening for specific problematic internet behaviors by using a new measure, the emergency department media use screener (EDMUS).

**Objective:**

The purpose of this study was to identify patterns of internet use in young people presenting with mental health concerns to the emergency department (ED), ascertain associations with their mental health, and evaluate whether the EDMUS can be used to predict subsequent ED presentations within 3 months.

**Methods:**

This cross-sectional retrospective study of Australian young people (N=149, aged 11-25 years; female: n=92, 61.7%) sought to use the EDMUS, a 24-item questionnaire, to identify problematic internet behaviors, including accessing or posting prosuicidal or proeating disorder content, cyberbullying, and inappropriate digital content. Data on each person’s mental health were extracted from electronic medical records to look for associations with EDMUS responses and ED re-presentation over 3 months. EDMUS items were grouped into clusters for analysis using chi-square tests, binary logistic regression, and path analyses.

**Results:**

Sharing suicidal digital content was the most common problematic internet use pattern identified by the EDMUS. However, this did not correlate with having a prior mental health diagnosis or predict readmission. Most participants had families with a concern for their internet use; however, this was less likely in participants with a diagnosis of personality disorder. Diagnoses of personality disorder or posttraumatic stress disorder were independent predictors of readmission (*P=*.003; *P=*.048).

**Conclusions:**

Although a history of complex psychopathology increases the likelihood of subsequent ED presentations, its links to internet use–related behaviors are still unclear. The EDMUS has potential for identifying young people who are most vulnerable to problematic internet behaviors and offers the opportunity for early intervention and potential prevention of more entrenched difficulties.

## Introduction

### Background

Internet use is continually rising, with around 27,000 new internet users every hour [1] and over 4.6 billion active users globally [2]. Around 89% of Australia’s population is on the internet today, compared to 76% in 2010 [3], with a majority of internet users aged 15-17 years [4]. There has been a 13% rise in mental illness in the past decade [5], with suicide being the leading cause of adolescent death in Australia [6], and the pandemic has further exacerbated the increase in child and adolescent mental health presentations [7].

In 1996, Young [8] laid the groundwork to describe mental health pathology related to increasing internet use. This has since morphed into internet gaming disorder (*Diagnostic and Statistical Manual of Mental Disorders*, Fifth Edition; DSM-5) and gaming disorder in the International Classification of Diseases (ICD-11), with ongoing debate on the validity of these diagnoses [9-11]. Further research on such specific internet behaviors has been encouraged and would aid in their formal recognition and classification by diagnostic manuals [12].

While several researchers have criticized the term “internet addiction” for its narrowness in describing only risky behaviors displayed by internet users [13-17], other terms used in literature such as “excessive internet use,” “pathological internet use” [18,19], “problematic interactive media use” [20], and “compulsive internet use” [21] allude to the same notion of addiction. In the context of screening, this has resulted in measuring the frequency and length of internet use and its behavioral consequences, but not how time is spent digitally. As such, an individual spending excessive time watching educational videos could score the same as an individual gaming on the internet every day, despite these behaviors having different impacts and concerns. This paper defines problematic internet behaviors as “internet use that is excessive, impulsive, or risky in nature, with adverse consequences to mental, physical, emotional, social, and functional health,” in line with most frequently used definitions in current literature, but additionally investigates a range of internet behaviors likely to indicate different kinds of mental health risk [22].

Accessing harm-advocating websites is one avenue through which excessive internet use seems to increase mental distress, while other problematic internet behaviors, such as excessive social media use, have been associated with increasing body image concerns and disordered eating [23-25]. Similarly, internet gaming disorder has been correlated with anxiety, depression, attention-deficit/hyperactivity disorder (ADHD) or hyperactivity symptoms, social phobia or anxiety, and obsessive-compulsive symptoms [26-28]. Furthermore, digital platforms provide opportunities for cyberbullying and sending inappropriate or unwanted content, leading to psychological distress [29].

Screen use can contribute toward increased risk in numerous ways. Cyberbullying, exposure to suicide-related content, problematic internet use, sexting, and frequency of social media use have all been associated with self-injurious thoughts and behaviors [30]. Recent social phenomena such as the Blue Whale Challenge and 13 Reasons Why have also contributed to normalizing or glorifying suicide. One study showed a substantial increase in self-harm emergency department (ED) visits among adolescents associated with the release of 13 Reasons Why [31]. Yet, screening for such problematic internet behaviors is not routine practice and occurs broadly in the context of internet addiction through monitoring screen time or withdrawal behaviors [32,33].

While rates of mental health presentations to the ED in adolescents are increasing in New South Wales, the prevalence of internet-related presentations remains unclear [7,34]. One study of over 200 adolescents hospitalized for psychiatric reasons showed that over 20% responded that their admission was related to something that happened on the internet [35]. A limited number of studies have discussed internet use in the context of EDs, while research on youth hospitalized or within the community is more common [36]. This study was proposed after a cluster of suicides in adolescents was noted in various areas of a large metropolitan city in Australia, with the use of social media to access suicide-related material on the internet being at the forefront of these presentations.

### The Emergency Department Media Use Screener

Despite evidence linking excessive internet use with mental illness, psychiatrists generally have a poor understanding of their patient’s internet use [37]. Hence, researches supporting screening tools are critical in assessing risk and implementing appropriate supports to minimize harm from problematic internet behaviors and are vital next steps in addressing this knowledge gap [38-40].

**Figure 1:**
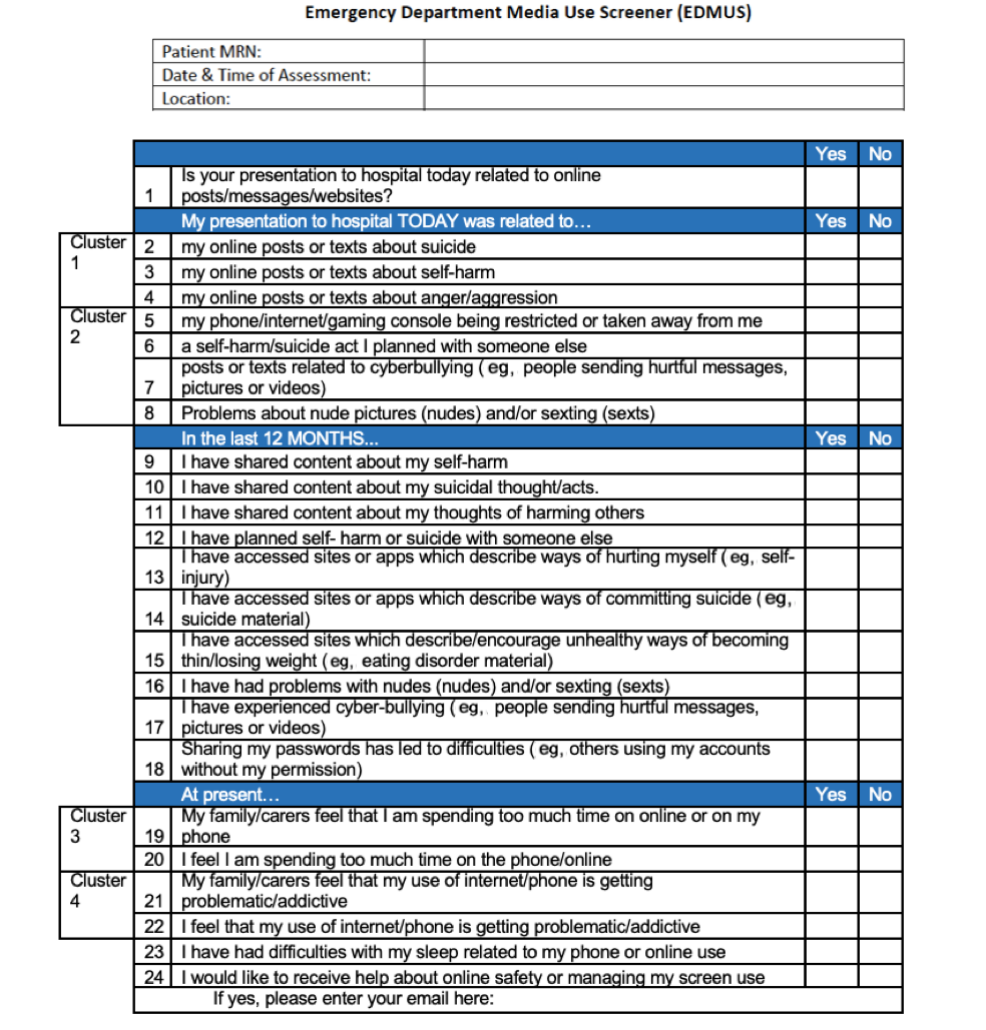
The emergency department media use screener (EDMUS).

The emergency department media use screener (EDMUS) is a screening tool developed by clinicians in 2019 and is being piloted as part of a quality improvement project for use in the Campbelltown Hospital ED in Sydney (Figure 1). The EDMUS was developed in response to increasing ED presentations of suicides facilitated by the internet through the sharing of news, apps, and content among adolescents [41,42]. Hence, the tool has a heavy focus on suicide and self–harm-related items. Adolescents presenting to the ED with mental health concerns were also screened for other specific internet behaviors that may indicate risk of psychopathology, including aggression, accessing proeating disorder websites, and cyberbullying, through 24 yes-or-no items assessed digitally.

### Research Objectives and Questions

The study had 3 primary research questions concerning adolescents aged 11-25 years. They were as follows:

1. What are the rates of participant responses to each EDMUS item?
2. Do EDMUS responses vary depending on an individual’s mental health diagnosis?
3. Do EDMUS responses and mental health diagnoses predict future ED presentations?

The following were the study objectives:

- Screening for patterns of media use in young people presenting to the ED with a mental health concern.
- Ascertaining whether patterns of media use vary depending on the mental health backgrounds of individuals.
- Deciphering whether patterns of media use can predict subsequent ED presentations.

## Methods

### Study Design

Campbelltown Hospital is located on the south-west fringe of metropolitan Sydney and has one of the highest rates of ED presentations for mental health concerns across this age group in the state of New South Wales, Australia. A cross-sectional retrospective study design was adopted. A web-based survey was distributed to young people aged 11-25 years presenting to the Campbelltown ED with a mental health concern from August 2020 to March 2021. Upon completion, the survey generated helpful links for young people to use. Responses were collected via the University of New South Wales’ Research Electronic Data Capture (REDCap), a secure web-based data management and survey tool [43,44]. This tool was linked to patients’ medical records to extract demographic and diagnostic data.

The inclusion criteria of the study included young people aged between 11 and 25 years who completed an EDMUS screen upon presenting to the Campbelltown Hospital ED with a mental health concern. The exclusion criteria included young people who presented to the Campbelltown Hospital ED, completed an EDMUS screen, but had a medical record number that was invalid or could not be ascertained, and young people outside the age range of the study.

### Measurements

#### Demographics

Demographic and clinical characteristics, including age, gender, educational status, Indigenous status, culturally and linguistically diverse status, postcode, ethnicity, date and time of arrival and departure at the ED, presenting problem, mode of arrival, mental health diagnoses, discharge diagnoses, and referrals, were investigated.

#### Problematic Internet Behaviors—EDMUS

The EDMUS was designed with a clinical purpose and not with the intent of assigning scores to items. Hence, the total EDMUS score was not a metric of choice. Tetrachoric correlation matrices (Multimedia Appendices 1 and 2) and dendrograms (Multimedia Appendix 3) were constructed to assess multicollinearity between variables. EDMUS items that most highly correlated with each other were grouped into clusters to test research questions 2 and 3. Cluster 1 included EDMUS items 2,3, and 4; cluster 2 included EDMUS items 5,6,7, and 8; cluster 3 included EDMUS items 19 and 21; and cluster 4 included EDMUS items 20 and 22 (Figure 1). A “yes” was assigned to a cluster if participants answered “yes” to any of the items within the cluster. Items 9-18 were omitted from further analysis as inaccurate recall was deemed to reduce the reliability of responses, given that participants were asked to recall events that occurred 12 months prior to the presentation [45-48].

#### Mental Health Status

Mental health diagnoses from medical records were grouped for ease of data analysis, following DSM-5 groupings. In particular, anxiety disorders (n=57) comprised participants with generalized anxiety disorder (n=55) and social anxiety (n=4). Personality disorders (PDs; n=39) comprised those who had cluster B traits (n=12), cluster C traits (n=3), antisocial PDs (n=3), and borderline PDs (n=28). Only mental health diagnoses with a sample size above 12 were considered for further analysis, representing at least 10% of those with mental health diagnoses in our sample. Multimedia Appendix 4 lists the range of mental health diagnoses in our sample.

### Statistical Analysis

Research question 1 was tested through a descriptive analysis of participant responses to each EDMUS item. Age and gender differences for each EDMUS item were also evaluated. Participants were split into 3 age groups, from youngest to oldest in equal increments for ease of analysis: 11-15, 16-20, and 21-25 years of age.

Research question 2 was tested by performing a chi-square analysis to test for differences in EDMUS responses across different diagnostic categories for mental health and associated concerns. *P* values from the Fisher exact test were used for analyses with a sample size of less than 5 participants.

Research question 3 was tested by examining associations between EDMUS clusters and subsequent ED presentations 3 months after their initial visit. Statistically significant clusters and mental health diagnoses identified through chi-square analysis were stratified through a binary logistic regression model. This analysis aimed to evaluate the association with readmission (defined as re-presenting to the ED within 3 months of a recorded visit) after adjusting for confounders. A structured equation model path analysis was constructed as a final model showcasing the relationship between statistically significant findings.

Statistical analyses were conducted on IBM SPSS Statistics 26 (IBM Corp) [49] and Stata (StataCorp LLC) [50]. For statistical analyses, a *P* value <.05 was considered statistically significant. Given the correlation of the variables with each other, correcting the *P* value based on the number of comparisons would have increased the type-2 error. Therefore, multitesting corrections for this proof-of-concept study were not conducted. After the extraction of relevant data from medical records and coding for analysis, there were 13 missing values. Since these comprised less than 5% of the total number of cases, listwise deletion was considered appropriate.

### Ethics Approval

Ethical approval was obtained from the Human Research Ethics Committee, South West Sydney Local Health District (2021/ETH00321). Patients completed the measure as part of a quality-improvement project rolled out internally by the hospital, and their data was shared with researchers by the data custodian as approved by the district’s ethics office. This did not involve consent by individual clients but rather consent from the institution for the use of their data, which was obtained.

## Results

### Respondents’ Characteristics

Baseline demographics of participants are presented in Table 1.

**Table 1:** Demographics of respondents included in the study (N=149).

Demographic		Value
Age (years), mean (SD); range		17.31 (3.387); 11-25
**Age group, n (%)**		
	11-15 years	55 (36.9)
	16-20 years	64 (42.3)
	21-25 years	30 (20.1)
**Gender, n (%)**		
	Male	56 (37.6)
	Female	92 (61.7)
	Other	1 (0.7)
**Age group by gender, n (%)**		
	11-15 years, males	13 (8.72)
	11-15 years, females	42 (28.2)
	11-15 years, other	0 (0)
	16-20 years, males	28 (18.8)
	16-20 years, females	35 (23.5)
	16-20 years, other	1 (0.67)
	21-25 years, males	15 (10.1)
	21-25 years, females	15 (10.1)
	21-25 years, other	0 (0)
**Indigenous status, n (%)**		
	Aboriginal	34 (22.8)
	Torres Strait	1 (0.70)
	Neither	114 (76.5)
**CALD^a^, n (%)**		
	Yes	2 (1.30)
	No	147 (98.7)
Decile (IRSD^b^)		4.89
Mean (SD)		2.689
**At least 1 psychiatric diagnosis, n (%)**		
	Yes	120 (80.54)
	No	29 (19.46)
	Cluster 1 (yes)	18 (12.1)
	Cluster 2 (yes)	18 (12.1)
	Cluster 3 (yes)	37 (24.8)
	Cluster 4 (yes)	32 (21.5)
**More than 1** **mental health** **diagnosis**		
	Yes	98 (65.8)
	No	51 (34.2)
	Cluster 1 (yes)	14 (9.40)
	Cluster 2 (yes)	15 (10.1)
	Cluster 3 (yes)	31 (20.8)
	Cluster 4 (yes)	35 (23.5)

^a^CALD: culturally and linguistically diverse.

^b^IRSD: index of relative socio-economic disadvantage.

**Figure 2:**
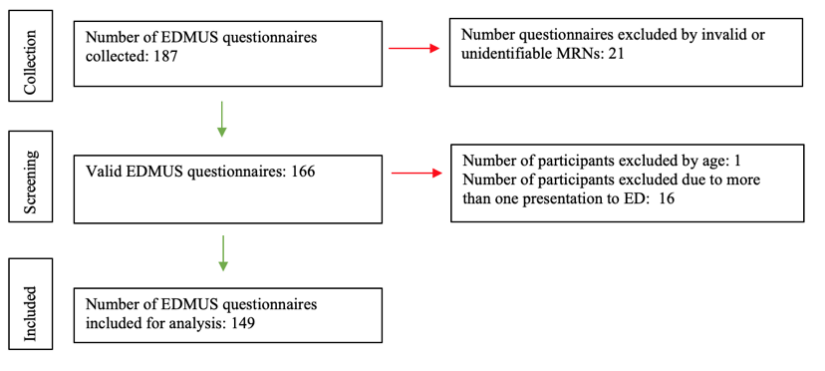
Recruitment flowchart. ED: emergency department; EDMUS: emergency department media use screener; MRN: medical record number.

A total of 149 participants met the study criteria and were included in the analysis (Figure 2). Participants had a mean age of 17.3 (SD 3.39; range 11-25) years at the time of EDMUS screening, were mostly female (n=92, 61.7%), not from an Indigenous background (n=114, 76.5%), and presented predominantly with suicide- or self-harm–related concerns and behaviors (n=118, 79.2%). Among participants, 80.5% (n=120) had a mental health diagnosis, of which depression (n=82, 55.0%) and anxiety (n=57, 38.3%) were most common.

### Analysis 1: Rates of EDMUS Responses

Table 2 summarizes EDMUS response rates by age and gender. EDMUS item 19 (family concern) had the highest endorsement, with 50 of 149 (33.6%) participants responding “yes,” followed by item 10 (sharing suicidal content on the internet in the past 12 months; n=43, 28.9%) and item 20 (own concern about internet use; n=39, 26.2%). Item 8 (experiencing problems with inappropriate, unwanted content) had the lowest “yes” response rate, with 5 of 149 (3.4%) participants responding “yes.”

Among the 3 age groups, participants aged 11-15 and 16-20 years were most likely to endorse items. Female participants affirmed EDMUS items more than male participants. Across male and female participants, item 19 (family concern) had the highest response rate in the age group of 11-15 years (63.6%, n=35). Response rates decreased with age across genders, except for cluster 2, which had peak response rates in the age group of 21-25 years.

**Table 2:** Emergency department media use screener (EDMUS) response rates (%) by age and gender.

		Gender (yes), %													Total (yes), %					Total population (yes), %
		Female						Male												
		Age group (years)				Total		Age group (years)				Total		Age group (years)						
		11 to 15	16 to 20	21 to 25			11 to 15		16 to 20	21 to 25			11 to 15			16 to 20	21 to 25			
**EDMUS^a^ items**																				
	1	14.63	20.00	20.00	17.58		30.77		17.86	6.67	17.86		18.52			18.75	13.33	17.4		
	2	11.90	11.43	13.33	11.96		15.38		10.71	0	8.93		12.73			10.94	6.67	10.7		
	3	14.29	8.57	13.33	11.96		30.77		10.71	0	12.50		18.18			9.38	6.67	12.1		
	4	14.29	17.14	6.67	14.13		30.77		7.14	0	10.91		18.18			12.50	3.45	12.8		
	5	14.29	0	6.67	7.61		23.08		0	0	5.36		16.36			0	3.33	6.70		
	6	11.90	8.57	6.67	9.78		15.38		3.57	0	5.36		12.73			6.25	3.33	8.10		
	7	9.76	5.71	6.67	7.69		15.38		0	6.67	5.36		11.11			3.13	6.67	6.70		
	8	9.76	0	6.67	5.49		0		0	0	0		7.41			0	3.33	3.40		
	9	38.10	28.57	13.33	30.43		30.77		17.86	0	16.07		36.36			23.44	6.67	24.8		
	10	42.86	31.43	26.67	35.87		23.08		25.00	0	17.86		38.18			28.13	13.33	28.9		
	11	14.29	5.88	13.33	10.99		0		7.14	0	3.57		10.91			6.35	6.67	8.10		
	12	11.90	8.57	6.67	9.78		0		3.57	0	1.79		9.09			6.25	3.33	6.70		
	13	16.67	14.29	0	13.04		15.38		7.14	0	7.14		16.36			10.94	0	10.7		
	14	21.43	11.43	6.67	15.22		7.69		10.71	0	7.14		18.18			10.94	3.33	12.1		
	15	23.81	11.43	0	15.22		7.69		7.14	6.67	7.14		20.00			9.38	3.33	12.1		
	16	9.52	14.29	7.14	10.99		7.69		7.14	6.67	7.14		9.09			10.94	6.90	9.40		
	17	38.10	22.86	6.67	27.17		30.77		10.71	6.67	14.29		36.36			17.19	6.67	22.1		
	18	9.52	2.86	0	5.43		7.69		0	6.67	3.57		9.09			1.56	3.33	4.70		
	19	61.90	11.43	13.33	34.78		69.23		25.93	6.67	30.91		63.64			19.05	10.00	33.6		
	20	41.46	14.29	13.33	26.37		46.15		28.57	6.67	26.79		42.59			20.31	10.00	26.2		
	21	43.90	11.43	6.67	25.27		53.85		14.29	6.67	21.43		46.30			14.06	6.67	24.2		
	22	36.59	14.29	6.67	23.08		30.77		7.14	6.67	12.50		35.19			12.50	6.67	19.5		
	23	34.15	17.14	6.67	23.08		30.77		21.43	6.67	19.64		33.33			18.75	6.67	21.5		
	24	24.39	8.57	0	14.29		15.38		3.57	0	5.36		22.22			6.25	0	10.7		
**EDMUS clusters**																				
	1	23.8	20.0	17.4	20.7		38.5		10.7	0	14.3		27.3			15.6	6.67	18.1		
	2	31.0	11.4	10.7	21.7		38.5		3.60	6.67	12.5		34.0			7.81	13.3	18.1		
	3	66.7	14.3	12.1	38.0		69.2		25.0	6.67	30.4		67.3			20.3	10.0	35.6		
	4	45.2	20.0	12.8	30.4		46.2		28.6	0	26.8		46.3			25.0	10.0	29.5		

^a^EDMUS: emergency department media use screener.

### Analysis 2: Associations With Background Mental Health Diagnoses

#### Participant Well-being

The prevalence of mental health diagnoses in accordance with EDMUS clusters is summarized in Table 3. A total of 36 different mental health diagnoses were extracted from the medical records of participants (Multimedia Appendix 2). After grouping similar disorders into broader diagnostic categories and excluding diagnoses with fewer than 12 participants, 7 mental health categories remained. These were depression, anxiety disorders, PD, posttraumatic stress disorder (PTSD), ADHD, autism spectrum disorder, and oppositional defiant disorder.

**Table 3:** Emergency department media use screener (EDMUS) cluster responses and chi-square or Fisher exact test results by mental health history of participants.

EDMUS^a^ items (total “yes” responses)			Depression		Anxiety disorders		PD^b^		PTSD^c^		ADHD^d^		ASD^e^		ODD^f^
**Cluster 1 (n=27)**															
	Response rate, n (%)^g^	13 (15.9)		9 (15.8)		7 (17.9)		2 (7.14)		6 (21.4)		6 (27.3)		4 (25.0)	
	Chi-square or Fisher exact test (*P* value)	.43		.56		.97		.11		.61		.23		.49	
**Cluster 2 (n=27)**															
	Response rate, n (%)	13 (15.9)		13 (22.8)		6 (15.4)		6 (21.4)		7 (25.0)		2 (9.10)		3 (18.7)	
	Chi-square or Fisher exact test (*P* value)	.42		.27		.63		.60		.31		.37		1.00	
**Cluster 3 (n=53)**															
	Response rate, n (%)	30 (36.6)		24 (42.1)		8 (20.5)		7 (25.0)		11 (39.3)		9 (40.9)		7 (43.8)	
	Chi-square or Fisher exact test (*P* value)	.78		.19		.02^h^		27		.65		.57		.47	
**Cluster 4 (n=44)**															
	Response rate, n (%)	25 (30.5)		19 (33.3)		7 (17.9)		4 (14.3)		8 (28.6)		9 (40.9)		4 (25.0)	
	Chi-square or Fisher exact test (*P* value)	.82		.45		.06^h^		.07^h^		.88		.21		.78	
Total participants, n (%)			82 (55.0)		57 (38.3)		39 (26.2)		28 (18.8)		28 (18.8)		22 (14.8)		16 (10.7)

^a^EDMUS: emergency department media use screener.

^b^PD: personality disorder.

^c^PTSD: posttraumatic stress disorder.

^d^ADHD: attention-deficit/hyperactive disorder.

^e^ASD: autism spectrum disorder.

^f^ODD: oppositional defiant disorder.

^g^Percentage of total participants with each mental health diagnoses.

^h^Values considered for further analysis.

Among participants, 80.5% (n=120) had at least 1 psychiatric diagnosis and 65.8% (n=98) had more than 1 psychiatric diagnosis recorded, with depression being the most common (n=82, 55%), followed by anxiety disorders (n=57, 38.3%) and PD (n=39, 26.2%).

#### Chi-square Analysis

Table 3 presents chi-square analyses between each EDMUS cluster and mental health diagnoses. A statistically significant association between PD and cluster 3 (*P*=.02) was found.

### Analysis 3: Predicting Readmission

From our sample, 58 (38.9%) participants presented to the ED within 3 months of their initial EDMUS screen, of whom 53 (91.4%) presented with mental health–related concerns.

Table 4 reports the binary logistic regression models after stratifying significant mental health diagnoses and clusters. Participants without PD whose families were not concerned about their internet use (cluster 3) and participants without PTSD who were concerned about their own internet use (cluster 4) were more likely to present to an ED subsequently (*P=*.02; *P=*.05).

**Table 4:** Stratification of clusters and mental health diagnoses.

		B		Significance		Exp (B)	95% CI
**PD^a^ (no)^b^**							
	Cluster 1	–0.244	0.716		0.784		0.211-2.908
	Cluster 2	0.543	0.421		1.721		0.459-6.455
	Cluster 3	–1.551	0.024		0.212		0.055-0.813
	Cluster 4	0.760	0.226		2.139		0.624-7.332
**PD (yes)**							
	Cluster 1	–20.335	0.999		0		0
	Cluster 2	–20.004	0.999		0		0
	Cluster 3	–20.327	0.999		0		0
	Cluster 4	79.495	0.998		3.343E+34		0
**PTSD^c^ (no)^d^**							
	Cluster 1	–0.120	0.837		0.887		0.283-2.780
	Cluster 2	–0.457	0.517		0.633		0.159-2.520
	Cluster 3	–0.948	0.134		0.387		0.112-1.339
	Cluster 4	1.151	0.053		3.162		0.985-10.151
**PTSD (yes)**							
	Cluster 1	21.421	0.999		2.009E+9		0
	Cluster 2	–0.436	0.688		0.647		0.077-5.442
	Cluster 3	22.076	0.999		0		0
	Cluster 4	–0.132	1.000		0.876		0

^a^PD: personality disorder.

^b^Model statistics: the model significantly (*P*=.001) predicted readmission, capturing 54.6% (Nalgelkerke *R***^2^**=0.546) of the variance in the outcome. The Hosmer and Lemeshow test of goodness of fit was not significant (*P*≥.99) indicating an adequate fit to the data.

^c^PTSD: posttraumatic disorder.

^d^Model statistics: The model significantly (*P*=.03) predicted readmission, capturing 42.0% (Nalgelkerke *R*^2^=0.420) of the variance in the outcome. The Hosmer and Lemeshow test of goodness of fit was not significant (*P*=.38) indicating an adequate fit to the data.

**Figure 3:**
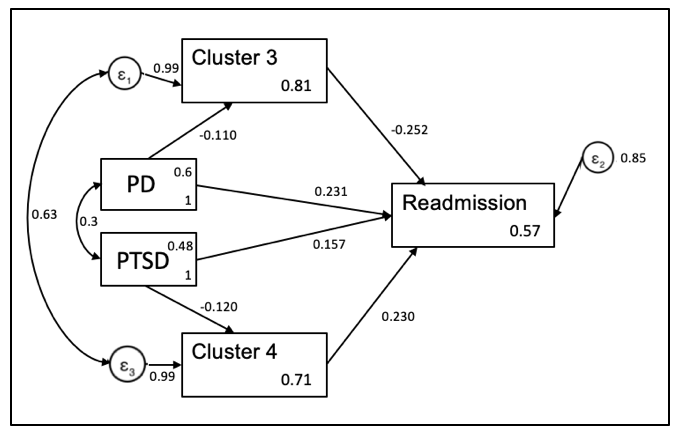
Structured equation model path analysis. PD: personality disorder; PTSD: posttraumatic disorder.

The path analysis (Figure 3, Table 5) revealed that cluster 3 (*P=*.009), cluster 4 (*P=*.02), PD (*P=*.003), and PTSD (*P=*.048) were independent predictors for readmission.

The arrows in Figure 3 suggest directions of associations with variables. The B coefficient suggests the degree of change in the outcome. A positive B coefficient suggests that an increase in the predictor variable increases the likelihood of the outcome, whereas a negative B coefficient suggests that an increase in the predictor variable decreases the likelihood of the outcome (eg, a participant with PD is less likely to answer “yes” to cluster 3).

**Table 5:** Structured equation model path analysis.^a^

	B	*P* value	95% CI
PD^b^ → Cluster 3	–0.11	.07	–0.238 to 0.019
Cluster 3	–0.25	.009	–0.441 to –0.062
Cluster 4	0.23	.02	0.039 to 0.419
PD	0.23	.003	0.077 to 0.385
PTSD^c^ → Readmission	0.16	.048	0.001 to 0.313
PTSD → Cluster 4	–0.12	.07	–0.247 to 0.008

^a^Model statistics: *χ*^2^_2_=2.661, *P*=.26, suggesting a good fit of the model to the data. The root mean squared error values were <0.05, indicating a close fit of the model to the data, and the Comparative Fit Index and Tucker-Lewis index values were >0.95, indicating a very good fit of the model to the data.

^b^PD: personality disorder.

^c^PTSD: posttraumatic stress disorder.

## Discussion

### Principal Findings

From our sample, 17.4% (n=26) presented to the ED with an internet use–related presentation. The EDMUS did not correlate with any mental health diagnosis. However, having a recorded diagnosis of PD or PTSD were strong independent predictors of readmission. While studies endorse the idea that having a PD is a predictor of readmission [51-53], our finding that PTSD is a readmission predictor contradicts the scarce literature [54].

### EDMUS Item Endorsement

Participants aged 11-15 years were more likely to endorse EDMUS items. Prior studies have demonstrated that younger adolescents are more likely to engage in harm-advocating digital content [55,56], while older age groups are more likely to post about positive experiences on the internet [57]. Some studies have also revealed that while older teenagers are more likely to use social media [58,59] and have more prevalent digital exposure to self-harm or suicide content [55], younger adolescents are more likely to reflect negatively about their negative digital experiences [59] and hence may be more likely to report them. This suggests that younger adolescents are more vulnerable to harmful digital content and may exhibit problematic internet behaviors that can be identified through internet use screening.

Female participants were more likely to endorse EDMUS items compared to male participants. Previous findings suggest that female participants are more likely to use social media and to have a negative digital experience [57]. Female participants are also more likely to engage in self-harm, with self-harm presentations in female participants aged 10-24 years almost twice that of young male participants of the same age [60]. Interestingly, cluster 2 was highly endorsed by female participants, except in the age group of 11-15 years. Our data showed higher “yes” responses among female participants, especially for item 8 (experiencing problems with inappropriate, unwanted content), endorsed by 5.49% (n=5) of female participants but by no male participants. This may be due to female participants being more likely to report negative digital experiences [59], including cyberbullying, and be negatively impacted by them [58,61]. However, it could also be the effect of having more female participants in this sample. Nevertheless, it is important to note such gender differences while screening, considering that low response rates by male participants may not necessarily reflect their intact mental well-being and may be a result of a social desire to be perceived as such.

Among the clusters investigated, cluster 3 had the highest endorsement, especially among younger participants. This is supported by literature indicating that parental concern and awareness of web-based activity decrease with age [58]. However, high response rates among younger participants may also be the effect of having a lower proportion of 21-25–year-olds in our sample compared to 11-15 and 16-20-year-olds. Nevertheless, familial awareness of internet behaviors would be valuable in various aspects, regardless of age, by mitigating risky behaviors early and providing monitoring and support, including access to interventions, and possibly improving health outcomes.

### Associations With Background Mental Health Diagnoses

Overall, “yes” responses for EDMUS items were generally low. There were statistically significant associations between cluster 3 and PD. This may have been the influence of selection bias due to the mental health of participants at screening. Given that in our sample, 79% (n=118) presented to the ED with suicidal ideation or attempt and 55% (n=82) had depression, it is likely that this biased the range of recorded mental health diagnoses in the sample toward internalizing presentations. Research suggests this association between internalizing behaviors as psychological difficulties and having negative self-schemas is likely to lead to higher web-based risk-taking [62], including methods of self-harm or suicide [56,63]. However, the low endorsement of EDMUS items in our sample may be a result of individual preferences not to disclose negative behaviors to preserve self-esteem. Given that data collection was completed during the COVID-19 pandemic, the effects of the lockdown may have impacted the mental health of participants and affected their EDMUS responses. While participants with depression may engage in more risky internet behavior, the influence of social desirability biases may result in lower “yes” response rates. Hence, any indication of poorer mental well-being should not be disregarded but rather explored further.

Participants with PD were less likely to answer yes to cluster 3 items about parental concern. Research on PD and its associations with parental concern and excessive internet use have been conducted in the context of internet addiction, using the scales mentioned previously [64-66]. Low parental concern was shown to increase the likelihood of internet addiction [67], possibly due to a lack of family support exacerbating the negative emotionality seen in individuals with PD and making them more susceptible to internet overuse [66,68]. This may be due to the challenges that come with the nature of the disorder itself, where families are less likely to get along with individuals with PD because of their personality characteristics. Emotional support from both parents would increase their child’s self-esteem, in turn reducing the risk of being addicted to the internet [65]. While parental concern was measured in the EDMUS, there is no indication of how this was perceived by participants—whether a low yes response was an indication of poor emotional support or as their internet use not being a cause for concern. Yet, previous research has found that 84% of people with PD retrospectively describe experiences of biparental neglect [67]. Hence, clinically, these findings may have some importance, as low EDMUS responses may be an indication of underlying psychopathology or poor social supports.

### Readmission

**Figure 4:**
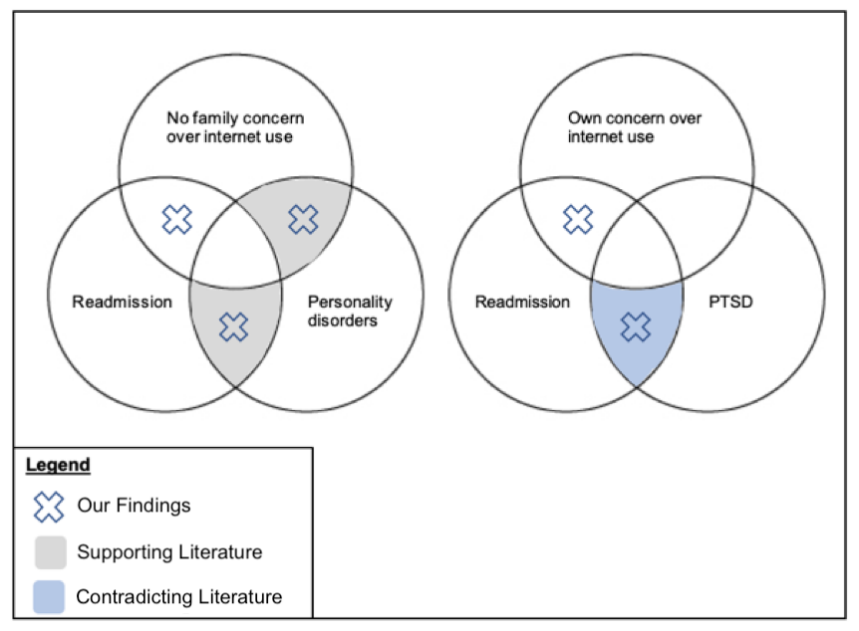
Venn diagrams summarizing our findings in the context of the current literature. PTSD: posttraumatic disorder.

Our final model suggests that while individuals with PD were more likely to re-present to the ED, their response to cluster 3 items was not statistically significant in predicting readmission. Individuals without PD and whose families had no concern about their internet use were more likely to re-present to the ED, suggesting that cluster 3 was a stronger independent predictor. Figure 4 summarizes these findings in the context of current literature. Studies on psychiatric diagnoses as possible predictors of readmission reveal increasing readmission rates associated with PD or affective disorders [51-53]. While research endorses that poor emotional support in general increases the likelihood of readmission, especially among individuals with psychiatric diagnoses [67,69], literature specific to family concern over internet use and readmission is lacking. Hence, these exploratory findings may address this knowledge gap. Similarly, the interaction between low family concern over internet use, readmission, and PD specifically has not been explored previously. While our findings suggest that these variables were independent predictors of readmission, further research on this specific interaction is needed to confirm our findings.

Further, a recorded history of PTSD was an independent predictor for readmission, contradicting the scarce emerging evidence. One meta-analysis on predictors of psychiatric readmission in children and adolescents found that while abuse and neglect were predictors for readmission in youth with externalizing disorders, having a history of PTSD was not a predictor for readmission [54]. While this study was done in the context of psychiatric in-patient readmission and in patients aged below 18 years, another study in the ED context in this age group seems to support their results [70]. It is possible that the recorded history of PTSD in our study may have reflected a history of abuse or neglect, which may have driven the statistical significance seen in our findings. Furthermore, participants in similar studies may have presented with PTSD and subsequently been readmitted, compared to primarily presenting with a depressive episode as in our study, with PTSD as a comorbidity. Nevertheless, given the degree of bias disclosed in studies within the meta-analysis, further research with larger sample sizes is required to clarify the relationships observed, including whether a history of complex psychopathology may increase the likelihood of a distressed individual re-presenting to the ED.

Our results suggest that posting about suicide was not predictive of readmission. This suggests that posting about suicide does not necessarily result in a suicidal act, contradicting previous research that has reported that accessing prosuicidal content increases the likelihood of suicidality [71]. However, recent studies question the usefulness of this information given the low validity of using search volumes to predict suicidal activity. For example, suicide-related terms used on the internet in Italy are more likely to be related to bereavement or curiosity than suicidality [72,73]. This highlights the need for clinical interviews in the future, as assumptions made through the detection of internet access may be flawed or misinterpreted.

Given that none of the other EDMUS clusters were predictive of readmission, this suggests how unpredictable mental health can be. Without follow-ups, it is difficult to know the rates of false-negatives and whether these had any impact on our findings. Hence, implementing screening or reviews at different time points may be valuable in assessing the risk posed by problematic internet behaviors for readmission and in identifying those individuals most at risk.

### Strengths and Limitations

To our knowledge, the EDMUS is one of the first tools to assess excessive internet use holistically, looking at patterns of behavior as opposed to a narrow focus on either internet addiction or suicide. This adds to its strength in raising valuable questions on excessive internet use and related behaviors. The EDMUS can provide clinicians with a better understanding of their patients’ behaviors and the internet’s underlying influence, hence potentially being a point of acute intervention for risk to others; for example, 6.7% (n=10) of our participants reported having planned self-harm or suicide with someone else. Furthermore, the EDMUS can be used as a means to facilitate conversations between patients and families, allowing for better matching of interventions and supports alongside implementation of risk-mitigating strategies.

Our sample had its strengths in being naturalistic, with participants seeking help, hence having high ecological validity. Our analysis was thorough, with the implementation of further tests to confirm our findings and provide valuable insights into risk profiles and problematic internet behaviors that can be incorporated clinically. The EDMUS generated links to help young people after discharge, in line with the recommendation for novel web-based help approaches from Biddle et al [36], adding to its strengths.

In hindsight, there were several modifications that would have strengthened the EDMUS and have been considered for future versions. Redesigning the EDMUS with appropriate recall intervals and using a Likert scale for a dimensional rather than categorical assessment would allow for a better measure of risk and improve the reliability of responses.

No causal relationships and directions of association can be established between variables due to the cross-sectional design of the study. In the future, having a larger sample size and conducting longitudinal studies would be valuable in exploring these associations further, especially between perceived and actual family support. The impact of the COVID-19 pandemic on the participants was not measured through the EDMUS and may have influenced an increase in mental health presentations to the ED and responses to the EDMUS.

### Conclusions

The EDMUS is a tool with potential for identifying problematic internet behaviors in young people. It also offers the opportunity for early intervention and the potential prevention of more entrenched difficulties. Identifying how young people access harm-advocating digital content may also be valuable in eliminating access and improving health outcomes. This study has reiterated that understanding a participant’s risk profile involves exploring their social supports, among other attributes. Assessing the usefulness of the EDMUS through follow-ups would be valuable in understanding the impact of its implementation.

## Appendix

Multimedia Appendix 1 Tetrachoric correlation matrix between each emergency department media use screener (EDMUS) item.

Multimedia Appendix 2 Tetrachoric correlation matrix between emergency department media use screener (EDMUS) clusters and mental health diagnosis.

Multimedia Appendix 3 Dendrogram to show collinearity between emergency department media use screener (EDMUS) items.

Multimedia Appendix 4 List of mental health diagnoses among participants.

## Abbreviations

ADHD: attention-deficit/hyperactive disorder
DSM-5: Diagnostic and Statistical Manual of Mental Disorders
ED: emergency department
EDMUS: emergency department media use screener
PD: personality disorder
PTSD: posttraumatic stress disorder

## Data Availability

The data for this project will not be made publicly available as it contains sensitive patient information. The ethics approval sought for the project did not include data sharing provisions. A limited subset of the data may be available upon request.

## References

[1] Roser M, Ritchie H, Ortiz-Ospina E Internet. Our World in Data.

[2] Johnson J (2021). Worldwide digital population as of January 2021. Statista.

[3] Individuals using the internet (% of population). The World Bank.

[4] Smart Jessica (2018). Digital technology use in the child, youth and family sector. Australian Institute of Family Studies.

[5] (2022). World mental health report: transforming mental health for all. World Health Organization.

[6] (2020). Suicide and self-harm monitoring. Australian Institute of Health Welfare.

[7] Hu N, Nassar N, Shrapnel J, Perkes I, Hodgins M, O'Leary F, Trudgett C, Eapen V, Woolfenden S, Knight K, Lingam R (2022). The impact of the COVID-19 pandemic on paediatric health service use within one year after the first pandemic outbreak in New South Wales Australia: a time series analysis. Lancet Reg Health West Pac.

[8] Young KS (1996). Psychology of computer use: XL. Addictive use of the Internet: a case that breaks the stereotype. Psychol Rep.

[9] Dullur P, Starcevic V (2018). Internet gaming disorder does not qualify as a mental disorder. Aust N Z J Psychiatry.

[10] King DL, Delfabbro PH, Potenza MN, Demetrovics Z, Billieux J, Brand M (2018). Internet gaming disorder should qualify as a mental disorder. Aust N Z J Psychiatry.

[11] van Rooij AJ, Ferguson CJ, Carras MC, Kardefelt-Winther D, Shi J, Aarseth E, Bean AM, Bergmark KH, Brus A, Coulson M, Deleuze J, Dullur P, Dunkels E, Edman J, Elson M, Etchells PJ, Fiskaali A, Granic I, Jansz J, Karlsen F, Kaye LK, Kirsh B, Lieberoth A, Markey P, Mills KL, Nielsen RKL, Orben A, Poulsen A, Prause N, Prax P, Quandt T, Schimmenti A, Starcevic V, Stutman G, Turner NE, van Looy J, Przybylski AK (2018). A weak scientific basis for gaming disorder: let us err on the side of caution. J Behav Addict.

[12] (2013). Diagnostic and statistical manual of mental disorders: DSM-5. American Academy of Paediatrics.

[13] Lachmann B, Duke É, Sariyska R, Montag C (2019). Who’s addicted to the smartphone and/or the internet?. Psychol Pop Media Cult.

[14] Laconi S, Rodgers RF, Chabrol H (2014). The measurement of internet addiction: a critical review of existing scales and their psychometric properties. Comput Human Behav.

[15] Widyanto L, Griffiths M (2006). ‘Internet addiction’: a critical review. Int J Ment Health Addiction.

[16] Yellowlees PM, Marks S (2007). Problematic internet use or internet addiction?. Comput Human Behav.

[17] Yau YHC, Crowley M, Mayes LM, Potenza MN (2012). Are internet use and video-game-playing addictive behaviors? Biological, clinical and public health implications for youths and adults. Minerva Psichiatr.

[18] Durkee T, Carli V, Floderus B, Wasserman C, Sarchiapone M, Apter A, Balazs J, Bobes J, Brunner R, Corcoran P, Cosman D, Haring C, Hoven C, Kaess M, Kahn J, Nemes B, Postuvan V, Saiz P, Värnik Peeter, Wasserman D (2016). Pathological internet use and risk-behaviors among European adolescents. Int J Environ Res Public Health.

[19] Fuchs M, Riedl D, Bock A, Rumpold G, Sevecke K (2018). Pathological internet use-an important comorbidity in child and adolescent psychiatry: prevalence and correlation patterns in a naturalistic sample of adolescent inpatients. Biomed Res Int.

[20] Nereim C, Bickham D, Rich M (2019). A primary care pediatricianʼs guide to assessing problematic interactive media use. Curr Opin Pediatr.

[21] Ciarrochi J, Parker P, Sahdra B, Marshall S, Jackson C, Gloster AT, Heaven P (2016). The development of compulsive internet use and mental health: a four-year study of adolescence. Dev Psychol.

[22] D'Angelo J, Moreno MA (2020). Screening for problematic internet use. Pediatrics.

[23] Fardouly J, Vartanian LR (2016). Social media and body image concerns: current research and future directions. Curr Opin Psychol.

[24] Holland G, Tiggemann M (2016). A systematic review of the impact of the use of social networking sites on body image and disordered eating outcomes. Body Image.

[25] Vannucci A, Flannery KM, Ohannessian CM (2017). Social media use and anxiety in emerging adults. J Affect Disord.

[26] Dullur P, Krishnan V, Diaz AM (2021). A systematic review on the intersection of attention-deficit hyperactivity disorder and gaming disorder. J Psychiatr Res.

[27] González-Bueso V, Santamaría JJ, Fernández D, Merino L, Montero E, Ribas J (2018). Association between internet gaming disorder or pathological video-game use and comorbid psychopathology: a comprehensive review. Int J Environ Res Public Health.

[28] King DL, Delfabbro PH (2016). The cognitive psychopathology of internet gaming disorder in adolescence. J Abnorm Child Psychol.

[29] Livingstone S, Stoilova M, Kelly A (2016). Cyberbullying: incidence, trends and consequences. Ending the Torment: Tackling Bullying from the Schoolyard to Cyberspace.

[30] Nesi J, Burke TA, Bettis AH, Kudinova AY, Thompson EC, MacPherson HA, Fox KA, Lawrence HR, Thomas SA, Wolff JC, Altemus MK, Soriano S, Liu RT (2021). Social media use and self-injurious thoughts and behaviors: a systematic review and meta-analysis. Clin Psychol Rev.

[31] Sinyor M, Mallia E, de Oliveira C, Schaffer A, Niederkrotenthaler T, Zaheer J, Mitchell R, Rudoler D, Kurdyak P (2022). Emergency department visits for self-harm in adolescents after release of the Netflix series '13 Reasons Why'. Aust N Z J Psychiatry.

[32] Bailey E, Rice S, Robinson J, Nedeljkovic M, Alvarez-Jimenez M (2018). Theoretical and empirical foundations of a novel online social networking intervention for youth suicide prevention: a conceptual review. J Affect Disord.

[33] Shafi RMA, Nakonezny PA, Romanowicz M, Nandakumar AL, Suarez L, Croarkin PE (2019). The differential impact of social media use on middle and high school students: a retrospective study. J Child Adolesc Psychopharmacol.

[34] Perera J, Wand T, Bein KJ, Chalkley D, Ivers R, Steinbeck KS, Shields R, Dinh M (2018). Presentations to NSW emergency departments with self-harm, suicidal ideation, or intentional poisoning, 2010-2014. Med J Aust.

[35] Gansner M, Belfort E, Cook B, Leahy C, Colon-Perez A, Mirda D, Carson N (2019). Problematic internet use and associated high-risk behavior in an adolescent clinical sample: results from a survey of psychiatrically hospitalized youth. Cyberpsychol Behav Soc Netw.

[36] Biddle L, Derges J, Goldsmith C, Donovan JL, Gunnell D (2018). Using the internet for suicide-related purposes: contrasting findings from young people in the community and self-harm patients admitted to hospital. PLoS One.

[37] Dullur P, Hay P (2017). Problem internet use and internet gaming disorder: a survey of health literacy among psychiatrists from Australia and New Zealand. Australas Psychiatry.

[38] Berryman C, Ferguson CJ, Negy C (2018). Social media use and mental health among young adults. Psychiatr Q.

[39] de Vries DA, Vossen HGM, van der Kolk-van der Boom P (2019). Social media and body dissatisfaction: investigating the attenuating role of positive parent-adolescent relationships. J Youth Adolesc.

[40] Shafi RMA, Romanowicz M, Croarkin PE (2018). #SwitchedOn: a call for assessing social media use of adolescents. Lancet Psychiatry.

[41] Ayers JW, Althouse BM, Leas EC, Dredze M, Allem J (2017). Internet searches for suicide following the release of 13 Reasons Why. JAMA Intern Med.

[42] Blue Whale: the game. Devon Children and Families Partnership.

[43] Harris PA, Taylor R, Thielke R, Payne J, Gonzalez N, Conde JG (2009). Research electronic data capture (REDCap): a metadata-driven methodology and workflow process for providing translational research informatics support. J Biomed Inform.

[44] Harris PA, Taylor R, Minor BL, Elliott V, Fernandez M, O'Neal L, McLeod L, Delacqua G, Delacqua F, Kirby J, Duda SN, REDCap Consortium (2019). The REDCap consortium: building an international community of software platform partners. J Biomed Inform.

[45] Schaeffer NC, Dykema J (2020). Advances in the science of asking questions. Annu Rev Sociol.

[46] Coughlin SS (1990). Recall bias in epidemiologic studies. J Clin Epidemiol.

[47] McPhail S, Haines T (2010). Response shift, recall bias and their effect on measuring change in health-related quality of life amongst older hospital patients. Health Qual Life Outcomes.

[48] Stopher P (2012). Collecting, Managing, and Assessing Data Using Sample Surveys.

[49] (2019). IBM support. IBM SPSS.

[50] (2021). Stata 17 released. The Stata Blog.

[51] Korkeila JA, Lehtinen V, Tuori T, Helenius H (1998). Frequently hospitalised psychiatric patients: a study of predictive factors. Soc Psychiatry Psychiatr Epidemiol.

[52] Mellesdal L, Mehlum L, Wentzel-Larsen T, Kroken R, Jørgensen HA (2010). Suicide risk and acute psychiatric readmissions: a prospective cohort study. Psychiatr Serv.

[53] Barker D, Jairam R, Rocca A, Goddard L, Matthey S (2010). Why do adolescents return to an acute psychiatric unit?. Australas Psychiatry.

[54] Edgcomb JB, Sorter M, Lorberg B, Zima BT (2020). Psychiatric readmission of children and adolescents: a systematic review and meta-analysis. Psychiatr Serv.

[55] Hawton K, Saunders KE, O'Connor RC (2012). Self-harm and suicide in adolescents. Lancet.

[56] Oksanen A, Näsi M, Minkkinen J, Keipi T, Kaakinen M, Räsänen P (2016). Young people who access harm-advocating online content: a four-country survey. Cyberpsychology.

[57] The digital lives of Aussie teens. Office of the eSafety Commissioner.

[58] Like, post, share: young Australians' experience of social media. Australian Communications and Media Authority.

[59] State of play-youth, kids and digital dangers. Office of the eSafety Commissioner.

[60] Sara G, Wu J, Uesi J, Jong N, Perkes I, Knight K, O'Leary F, Trudgett C, Bowden M (2023). Growth in emergency department self-harm or suicidal ideation presentations in young people: comparing trends before and since the COVID-19 first wave in New South Wales, Australia. Aust N Z J Psychiatry.

[61] Milton AC, Gill BA, Davenport TA, Dowling M, Burns JM, Hickie IB (2019). Sexting, web-based risks, and safety in two representative national samples of young australians: prevalence, perspectives, and predictors. JMIR Ment Health.

[62] Harris KM, McLean JP, Sheffield J (2009). Examining suicide-risk individuals who go online for suicide-related purposes. Arch Suicide Res.

[63] Livingston S, Haddon L, Görzig A (2012). Children, Risk and Safety on the Internet: Research and Policy Challenges in Comparative Perspective.

[64] Restrepo A, Scheininger T, Clucas J, Alexander L, Salum GA, Georgiades K, Paksarian D, Merikangas KR, Milham MP (2020). Problematic internet use in children and adolescents: associations with psychiatric disorders and impairment. BMC Psychiatry.

[65] Yao MZ, He J, Ko DM, Pang K (2014). The influence of personality, parental behaviors, and self-esteem on internet addiction: a study of Chinese college students. Cyberpsychol Behav Soc Netw.

[66] Zadra S, Bischof G, Besser B, Bischof A, Meyer C, John U, Rumpf H (2016). The association between internet addiction and personality disorders in a general population-based sample. J Behav Addict.

[67] Steele KR, Townsend ML, Grenyer BFS (2019). Parenting and personality disorder: an overview and meta-synthesis of systematic reviews. PLoS One.

[68] Lu WH, Lee KH, Ko CH, Hsiao RC, Hu HF, Yen CF (2017). Relationship between borderline personality symptoms and internet addiction: the mediating effects of mental health problems. J Behav Addict.

[69] Donisi V, Tedeschi F, Wahlbeck K, Haaramo P, Amaddeo F (2016). Pre-discharge factors predicting readmissions of psychiatric patients: a systematic review of the literature. BMC Psychiatry.

[70] Rosic T, Duncan L, Wang L, Eltorki M, Boyle M, Sassi R, Bennett K, Brotherston L, Pires P, Akintan O, Lipman E (2019). Trends and predictors of repeat mental health visits to a pediatric emergency department in Hamilton, Ontario. J Can Acad Child Adolesc Psychiatry.

[71] Tran US, Andel R, Niederkrotenthaler T, Till B, Ajdacic-Gross V, Voracek M (2017). Low validity of Google Trends for behavioral forecasting of national suicide rates. PLoS One.

[72] Ortiz P, Khin Khin E (2018). Traditional and new media's influence on suicidal behavior and contagion. Behav Sci Law.

[73] Solano P, Ustulin M, Pizzorno E, Vichi M, Pompili M, Serafini G, Amore M (2016). A Google-based approach for monitoring suicide risk. Psychiatry Res.

