# Supplementary material for: Screening for Media Use in the Emergency Department Among Young Australians: Cross-sectional Study
Source: JMIR Form Res. 2023 May 15;7:e42986. doi: 10.2196/42986 (PMC10227703; doi:10.2196/42986)
Supplement: Multimedia Appendix 1 [file formative_v7i1e42986_app1.docx]

Appendix 1: Tetrachoric correlation matrix between each EDMUS item.

|  |  | EDMUS item | | | | | | | | | | | | | |
| --- | --- | --- | --- | --- | --- | --- | --- | --- | --- | --- | --- | --- | --- | --- | --- |
|  | Readmission | 1 | 2 | 3 | 4 | 5 | 6 | 7 | 8 | 19 | 20 | 21 | 22 | 23 | 24 |
| Readmission | 1.00 | 0.11 | 0.09 | 0.29 | 0.03 | 0.32 | 0.04 | 0.31 | 0.31 | 0.26 | 0.00 | 0.24 | 0.04 | 0.17 | 0.09 |
| EDMUS item 1 | 0.11 | 1.00 | 0.47 | 0.73 | 0.83 | 0.07 | 0.19 | 0.80 | 0.69 | 0.33 | 0.38 | 0.17 | 0.41 | 0.43 | 0.33 |
| EDMUS item 2 | 0.09 | 0.47 | 1.00 | 0.99 | 0.86 | 0.51 | 0.22 | 0.65 | 0.31 | 0.49 | 0.61 | 0.48 | 0.49 | 0.53 | 0.44 |
| EDMUS item 3 | 0.29 | 0.73 | 0.99 | 1.00 | 0.59 | 0.54 | 0.39 | 0.59 | 0.35 | 0.70 | 0.40 | 0.50 | 0.48 | 0.60 | 0.56 |
| EDMUS item 4 | 0.03 | 0.83 | 0.86 | 0.59 | 1.00 | 0.59 | 0.35 | 0.81 | 0.23 | 0.50 | 0.48 | 0.60 | 0.56 | 0.71 | 0.48 |
| EDMUS item 5 | 0.32 | 0.07 | 0.51 | 0.54 | 0.59 | 1.00 | 0.75 | 0.50 | 0.75 | 1.00 | 0.42 | 0.81 | 0.41 | 0.19 | 0.30 |
| EDMUS item 6 | 0.04 | 0.19 | 0.22 | 0.39 | 0.35 | 0.75 | 1.00 | 0.43 | 0.41 | 0.41 | 0.14 | 0.19 | 0.39 | 0.08 | 0.36 |
| EDMUS item 7 | 0.31 | 0.80 | 0.65 | 0.59 | 0.81 | 0.50 | 0.43 | 1.00 | 0.23 | 0.44 | 0.27 | 0.71 | 0.56 | 0.84 | 0.67 |
| EDMUS item 8 | 0.31 | 0.69 | 0.31 | 0.35 | 0.23 | 0.75 | 0.41 | 0.23 | 1.00 | 0.83 | 0.70 | 0.01 | 0.08 | 0.15 | 0.10 |
| EDMUS item 19 | 0.26 | 0.33 | 0.49 | 0.70 | 0.50 | 1.00 | 0.41 | 0.44 | 0.83 | 1.00 | 0.77 | 0.94 | 0.82 | 0.64 | 0.40 |
| EDMUS item 20 | 0.00 | 0.38 | 0.61 | 0.40 | 0.48 | 0.42 | 0.14 | 0.27 | 0.70 | 0.77 | 1.00 | 0.74 | 0.89 | 0.81 | 0.80 |
| EDMUS item 21 | 0.24 | 0.17 | 0.48 | 0.50 | 0.60 | 0.81 | 0.19 | 0.71 | 0.01 | 0.94 | 0.74 | 1.00 | 0.83 | 0.69 | 0.56 |
| EDMUS item 22 | 0.04 | 0.41 | 0.49 | 0.48 | 0.56 | 0.41 | 0.39 | 0.56 | 0.08 | 0.82 | 0.89 | 0.83 | 1.00 | 0.80 | 0.87 |
| EDMUS item 23 | 0.17 | 0.43 | 0.53 | 0.60 | 0.71 | 0.19 | 0.08 | 0.84 | 0.15 | 0.64 | 0.81 | 0.69 | 0.80 | 1.00 | 0.79 |
| EDMUS item 24 | 0.09 | 0.33 | 0.44 | 0.56 | 0.48 | 0.30 | 0.36 | 0.67 | 0.10 | 0.40 | 0.80 | 0.56 | 0.87 | 0.79 | 1.00 |

Interpretation: values close to 0 (shaded blue) indicate that variables are independent of each other and values close to 1 (shaded red) indicate that variables are not independent of each other (i.e. collinearity between variables)
