# Supplementary material for: Screening for Media Use in the Emergency Department Among Young Australians: Cross-sectional Study
Source: JMIR Form Res. 2023 May 15;7:e42986. doi: 10.2196/42986 (PMC10227703; doi:10.2196/42986)
Supplement: Multimedia Appendix 2 [file formative_v7i1e42986_app2.docx]

Appendix 2: Tetrachoric correlation matrix between EDMUS Clusters and mental health diagnosis

Interpretation: values close to 0 (shaded blue) indicate that variables are independent of each other and values close to 1 (shaded red) indicate that variables are not independent of each other (i.e. collinearity between variables)
