# Supplementary figures and images for: Screening for Media Use in the Emergency Department Among Young Australians: Cross-sectional Study
Source: JMIR Form Res. 2023 May 15;7:e42986. doi: 10.2196/42986 (PMC10227703; doi:10.2196/42986)

## Appendix 3: Dendrogram to show collinearity between EDMUS items.


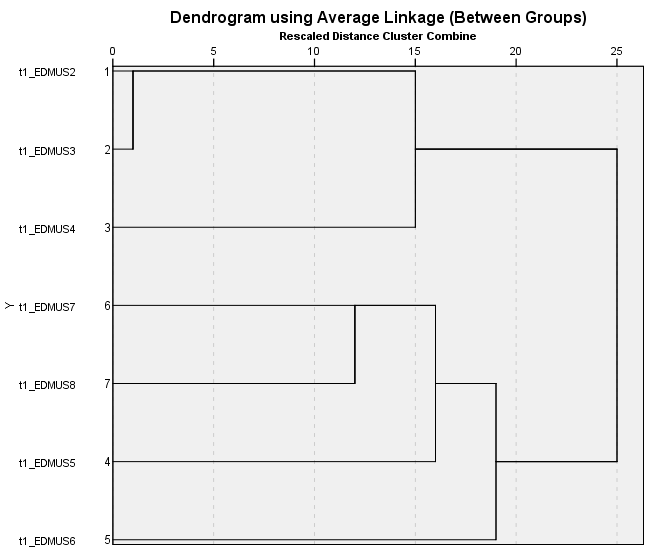

Supplement: Multimedia Appendix 3 [file formative_v7i1e42986_app3.docx]
