# Supplementary material for: Screening for Media Use in the Emergency Department Among Young Australians: Cross-sectional Study
Source: JMIR Form Res. 2023 May 15;7:e42986. doi: 10.2196/42986 (PMC10227703; doi:10.2196/42986)
Supplement: Multimedia Appendix 4 [file formative_v7i1e42986_app4.docx]

## Appendix 4: List of mental health diagnoses among participants.

| **Mental Health Diagnosis** | **Total Participants** | |
| --- | --- | --- |
|  | **(N)** | **(%)** |
| Cluster B traits | 12 | 8.05 |
| Cluster C traits | 3 | 2.01 |
| Borderline Personality Disorder | 28 | 18.8 |
| Antisocial Personality Disorder | 3 | 2.01 |
| Gaming Addiction | 2 | 1.34 |
| Social Anxiety Disorder | 4 | 2.68 |
| Anxiety Disorder | 55 | 36.9 |
| Major Depressive Disorder | 82 | 55 |
| Bipolar Disorder | 8 | 5.37 |
| Drug induced psychosis | 1 | 0.67 |
| PTSD | 28 | 18.8 |
| ADHD | 28 | 18.8 |
| ASD | 22 | 14.8 |
| Tourette Syndrome | 4 | 2.68 |
| OCD | 7 | 4.7 |
| ODD | 16 | 10.7 |
| DMDD | 3 | 2.01 |
| Schizophrenia | 7 | 4.7 |
| Epilepsy | 1 | 0.67 |
| Psychotic Disorder | 5 | 3.36 |
| Gender Identity Disorder | 1 | 0.67 |
| Intellectual Disability | 9 | 6.04 |
| learning disability | 2 | 1.34 |
| Dissociative Disorder | 2 | 1.34 |
| Dyslexia | 1 | 0.67 |
| Developmental Issues | 2 | 1.34 |
| Eating Disorder | 1 | 0.67 |
| Conversion Disorder | 1 | 0.67 |
| Depersonalisation Disorder | 1 | 0.67 |
